# Validation of the Persian Involuntary Musical Imagery Scale alongside multifaceted investigation of earworms among Iranian college students

**DOI:** 10.3389/fpsyg.2025.1480633

**Published:** 2025-01-31

**Authors:** Aref Tabahori, Imanollah Bigdeli, Hossein Kareshki

**Affiliations:** Department of Psychology, Ferdowsi University of Mashhad, Mashhad, Iran

**Keywords:** earworms, involuntary musical imagery, auditory perception, psychometric, individual differences, music cognition, music psychology

## Abstract

**Introduction:**

Involuntary musical imagery is considered a ubiquitous phenomenon worldwide, while mixed and inconsistent results regarding different aspects of earworms remain challenging. Furthermore, there is a special need for research on earworms in societies other than in European or American. The primary aim of our study is to validate the Persian Involuntary Musical Imagery Scale (PIMIS) and, secondly, to carry out a detailed exploration of earworms among Iranian college students.

**Methods:**

A total of 946 Iranian college students were recruited to answer the Persian Involuntary Musical Imagery Scale (PIMIS), and gold standards including the Thought Control Questionnaire (TCQ, to convergent validity), the Depression, Anxiety, and Stress Scale (DASS-21, to concurrent validity), the White Bear Suppression Inventory (WBSI, to predictive validity), as well as the NEO Five-Factor Inventory (NEO-FFI), the Obsessive-Compulsive Inventory-revised (OCI-R), and socio-demographic queries. A complex of features regarding the validity and reliability of the PIMIS, along with numerous aspects of earworm, were explored.

**Results and discussion:**

The Persian Involuntary Musical Imagery Scale encompasses 15 items along with three additional questions. It was found to be a valid and reliable instrument among Iranian college students, qualified to measure individual differences in earworms. Phenomenological evidence and detailed data on individual differences could provide rich knowledge for the rest of the literature paradigms. Moreover, future investigations into the local regions of the Iranian population are recommended.

## 1 Introduction

### 1.1 Background

One mysterious human talent is the ability to hear in his cognitive ear. Auditory imagery, especially musical imagery, has attracted more researchers in recent years (Küssner et al., 2022). Involuntary Musical Imagery (INMI, or earworms) is an ephemeral piece of music that pops up in someone’s mind involuntarily and then replays out of volition (Floridou et al., 2015). Many studies have indicated that INMI is an ordinary part of human life around the world (Bailes, 2007; Liikkanen, 2012b; Liikkanen et al., 2015) that may be the most frequent type of spontaneous cognition (Beaman and Williams, 2010).

INMI has been investigated in a wide variety of studies, from retrospective surveys (Liikkanen, 2008; Liikkanen, 2012b) and the experience-sampling method (Cotter and Silvia, 2017) to musical imagery induction (Liikkanen, 2012a; Floridou et al., 2018) and neural correlates exploration (Farrugia et al., 2015; Izadifar et al., 2023). Since retrospective self-reporting provides standardized findings in phenomenological studies of INMI (Cotter et al., 2016; Floridou et al., 2015), this methodology has been numerously employed in the investigation of earworm experiences (Liikkanen and Jakubowski, 2020). The Involuntary Musical Imagery Scale (IMIS) is a self-report tool developed by Floridou et al. (2015) to measure experiences of earworms. The IMIS thus far has been validated in Chinese (Jue et al., 2022) and African cultures (Pitman et al., 2023), which in both revealed strong psychometric properties.

However, the phenomenology of INMI is a relatively novel area of research (Liikkanen and Jakubowski, 2020), and previous literature points out insufficient data from non-Euro-American cultures (Pitman et al., 2023). In the-largest-sample study conducted around the world by Liikkanen et al. (2015), only data on earworms in Twitter messages in English were analyzed (biased against the non-English population), while 46 contributions were reported from Iran. Moreover, accumulating literature on INMI research in 21st century (Liikkanen, 2018) implies the major issue of contradictory results in this area which highlights a special need for “replication” and “reproduction” of certain findings in various cultures (Liikkanen and Jakubowski, 2020). These findings are addressed in subsequent sections regarding socio-demographics, personality traits, and the Asian context.

### 1.2 INMI and socio-demographics

Although a set of socio-demographics were numerously studied in relation to INMI, the literature does lack of consistency and/or cross-cultural replication.

For instance, gender was shown to be inconsistently related to INMI experience. Although some studies have demonstrated that females experience more earworms than males (Liikkanen, 2012b; Floridou et al., 2015), others have found no gender effect (Beaman and Williams, 2010; Jue et al., 2022; Beaman and Williams, 2013). Moreover, research has revealed that females experience longer earworm episodes than males (Pitman et al., 2023; Liikkanen, 2012a; Campbell and Margulis, 2015), while another study showed no such difference (Moeck et al., 2018).

Age was another factor involved in conflicting studies of INMI. Bailes (2015) found a positive relationship between age and earworm frequency, while others explored the negative correlation of age with frequency (Bennett, 2003; Liikkanen, 2012b; Floridou et al., 2019) and earworms section length (Pitman et al., 2023). Age was also found to be negatively correlated with the interference caused by earworms (Beaman and Williams, 2010).

Moreover, individuals’ states of cognitive load were another factor emerged differently across multiple studies. Hyman et al. (2013) believed earworms are more frequent during states of low and high cognitive load, whereas other studies have demonstrated that frequency of earworms rises as cognitive load diminishes (Floridou et al., 2017; Floridou and Mullensiefen, 2015; Liikkanen, 2012b). This concept has not been explored in the real context as well as in Asian societies.

On the other hand, evidence on the relationship between videogaming background and INMI experience in the Asian countries is lacking. A history of involvement in videogames is associated with a higher frequency of earworms (Floridou et al., 2022), and gamers also come up with more involuntary visual and musical imagery (Ortiz De Gortari, 2018), which can help play videogames (Achtman et al., 2008). However, this issue needs to be sufficiently focused on in the literature (Floridou et al., 2022).

Furthermore, studies exploring musicality and INMI has yielded divergent outcomes. Studies show that music students experience more earworms than others (Beaty et al., 2013; Hyman et al., 2013). Furthermore, musicians experience more frequent (Levitin, 2006; Liikkanen, 2012b), and intrusive earworms (Hyman et al., 2013) than non-musicians. However, other research indicated no difference between musicians and non-musicians (Beaman and Williams, 2010; Pitman et al., 2023; Floridou et al., 2015; Mullensiefen et al., 2014), between participants with and without musical training (Jue et al., 2022). Some have also obtained mixed results (Floridou et al., 2015).

Inconsistent results have also been found in individuals’ reactions to earworms. Some researchers believe people across different geographical and linguistic locations tend to passively accept their earworms (Beaman and Williams, 2010; Williamson et al., 2014; Liikkanen and Jakubowski, 2020), showing earworms are pleasant experiences (Halpern and Bartlett, 2011; Liikkanen, 2012b; Liikkanen and Jakubowski, 2020; Beaman and Williams, 2010; Floridou et al., 2015). By contrast, some other researchers found earworms to be aversive (Liikkanen, 2008; Williamson et al., 2014). These conflicting ideas necessitate further studies (Pitman et al., 2023).

Another notable point for investigation is whether individuals primarily experience liked songs as INMI or disliked ones. Several studies state that earworms of liked music are more likely to be experienced than disliked tunes (Beaman and Williams, 2010; Liikkanen, 2012b; Hyman et al., 2015). However, no related results were explored in Asian countries.

Additionally, evidence on linguistic dimension of INMI experience is insufficient. Liikkanen (2012b) notes that language and lyrics are not essential factors in musical imagery. Nevertheless, no research was carried out in Asian countries.

Likewise, it seems that there are scarce data on recognizing earworms’ triggers. There is a consensus that INMI experiences often have recognizable triggers (Williamson et al., 2012; Williamson et al., 2014) with no replication in the Asian countries.

Findings regarding the frequency of INMI experiences in different cultures were notably discrepant. According to Liikkanen (2012b), almost 90% of Euro-Americans report experiencing earworms at least once a week, but Jue et al. (2022) suggested 49.3% at least once a week and 10.5% every day for the Chinese context. Furthermore, Pitman et al. (2023) reported 89.5% once a week or more and 31.5% daily in African culture.

Similarly, there is very little evidence on the relationship between home language and elements of INMI experiences. In an African sample, individuals whose home language is English report experiencing shorter sections of earworms than those whose home language is not English (Pitman et al., 2023). This result would be different in Asian population due to distinct language variety.

### 1.3 INMI and personality traits

The association between INMI and personality characteristics present a complicated picture, as different studies have reported varying results.

There is no consensus about the relationship between Big-Five personality factors and earworms. Cotter et al. (2016) suggest that people with higher scores on openness tend to experience more earworms, and Williamson and Müllensiefen (2012) mention that neurotic individuals report more earworms. However, other studies have demonstrated no correlation of openness and neuroticism with earworm frequency (Floridou et al., 2012; Jue et al., 2022). Moreover, Jue et al. (2022) found that open people to new experiences tend to have longer sections and episodes of earworms. They also found that neuroticism and openness correlate with the IMIS total score, that neurotic people are inclined to find their earworms unpleasant, and that openness is associated with a physical reaction to earworms.

There is a disagreement on the relationship between obsessive-compulsive traits and different aspects of earworms. Mullensiefen et al. (2014) found that higher OC traits coincide with more frequent and unpleasant earworms. Floridou et al. (2015) found that higher OC traits are only associated with an adverse reaction to earworms. Jue et al. (2022) revealed a correlation of OC traits with IMIS total score and its four factors but not with frequency and section length, which is considered a concern to be investigated in other Asian countries.

Furthermore, people careful with inner thoughts are more prone to frequent earworms (Bailes, 2015; Baruss and Wammes, 2009) whereas no studies have explored this experience in countries with religious background including Iran.

Previous literature considers two theoretical backgrounds for INMI as a type of unwanted thoughts: 1- Zeigarnik’s Effect (Zeigarnik, 1927) [e.g. *as a piece of music does start in our mind, it might get back to the mind again later* (Hyman et al., 2013)]; 2- Ironic Mental Control Theory (Wegner, 1994) [e.g. *as you try to quite a music in your mind, it might continue more*]. However, INMI was not investigated in relation with thought control techniques and/or inclination toward suppression of unwanted thoughts in Iranian context.

Some studies point out that INMI includes an emotional dimension (Williamson and Müllensiefen, 2012), and a study by Floridou et al. (2015) demonstrated that the IMIS help and negative valence was correlated, which reflects a “duality” of earworms’ usefulness-disturbance. Furthermore, Ulor et al. (2022) elaborated this finding to affective states, in which individuals with higher anxiety and depressive symptoms report that their earworms are not only frequent and aversive but also helpful. However, no study has been explored this association in Asian countries especially Iran.

### 1.4 INMI in the Asian context

In a Chinese sample, three findings for the first time have been revealed (Jue et al., 2022). (1) Urban-residing people experience more earworms than rural-residing ones. (2) The frequency of earworms in the only-child group is more than others with siblings. (3) The amount of time people listen to music every day affects the frequency of earworms. However, it would be important to be explored in another Asian country with distinct socio-cultural characteristics.

### 1.5 Objective

Overall, reproduction of the above research regarding INMI along with exploration of related constructs in Iranian socio-demographic context with a rich linguistic and cultural variety would be precious. To the best of our knowledge, there has not yet been an instrument to measure earworms in Persian culture, nor has any research been carried out in this field to explore phenomenological dimensions of earworms. Thus, the aim of the current study is to investigate psychometric properties of the Persian Involuntary Musical Imagery Scale (PIMIS) in Iranian context, which also helps to uncover various sides of earworms experience in this society.

## 2 Methods

Different instruments were applied in distinct phases, which paved the way for the investigation of related constructs without the respondents being overwhelmed. As the current research was divided into four phases (0–3), it would be considered a cross-sectional study.

### 2.1 Participants and procedures

The translation and validation processes were conducted according to the guideline suggested by Boateng et al. (2018) on the development and validation of instruments in behavioral sciences.

#### 2.1.1 Phase 0

The original IMIS was translated from English to Persian by three individuals: the first author of the current study, a bachelor’s and a master’s student of psychology who were native Persian speakers and proficient in English. After a comparison of forward translations, the revised version was translated back into English by a professional Persian-English translator unfamiliar with the subject area. The back-translated version was evaluated by a faculty member of psychology and a faculty member of linguistics proficient in English to verify face validity and compare conceptual vicinity with the original scale, respectively. Additionally, five faculty members from the Department of Psychology were requested to answer the final version of PIMIS items and rate them on a content validity form. Next, a pilot study was conducted on 15 participants who had experienced earworms, based on a three-stage cognitive interview: five bachelor’s students attended in the first session, five master’s students participated in the second session, and thirdly five PhD students volunteered. It took an average of 4 min to answer the items, and all participants stated that they recognized the earworm phenomenon well.

#### 2.1.2 Phase 1

Exploiting convenience sampling, 342 students from Ferdowsi University of Mashhad were recruited from 31 December 2023 to 23 January 2024. The questionnaires were built on the SHAHTA website, then an invite link was made to be shared through platforms such as Telegram groups and Short Message Service [SMS], or through printed QR codes which were distributed at any time of the day in locations such as classrooms, faculty halls, dormitories, and the university campus by the authors of the current research. All participants were informed on the earworms (whether online or on-site), and accepted the informed consent to take part in our study on the SHAHTA website. After careful initial screening for inattentiveness, accidental response, and not having experience with earworms, 17 cases (5%) were deleted. After an exploratory analysis, six participants were multivariate outliers and omitted with a trivial proportion (2%), so 319 participants (84 males, 235 females) remained that aged between 17 and 40 (*M* = 22.24, SD = 3.29; 9 missing). Then, data underwent a set of procedures including item analysis, exploratory factor structure, and internal reliability assessment. The exploited instruments were the PIMIS, socio-demographic questions, and one established instrument to explore the association between unwanted thought control techniques and earworm (for convergent validity of the PIMIS). By the way, the instruments were in the same order for all participants.

#### 2.1.3 Phase 2

Using convenience sampling, 320 students from Ferdowsi University of Mashhad were recruited from 17 February to 6 March 2024 with no invalid responses. The process of delivery to the participants were exactly the same as phase 1. Eight participants were multivariate outliers and dropped with a negligible rate (2.5%). So, 312 participants (57 males, 255 females) were retained that aged between 18 and 50 (*M* = 23.47, SD = 5.07; 5 missing). Ultimately, the data were used for internal reliability approval, and the factor structure confirmation. The utilized instruments were the PIMIS, socio-demographic questions, and one criterion to measure the relationship of depression and anxiety scores with earworm (for concurrent validity of the PIMIS). Moreover, the instruments were in the same order for all participants.

At least 3 weeks later (from 2 to 24 April 2024), 61 participants from phase 2 were selected for re-test. Then, they were requested to answer the PIMIS again (through a link sent by SMS as we had asked their mobile number at the end of phase 2). Two (3%) univariate outliers were eliminated, as well as seven (11%) cases with invalid responses, so that 52 participants were remained. The utilized instruments were the PIMIS along with a criterion to measure the correlation between attitude towards unwanted thought suppression and earworm (for predictive validity of the PIMIS). Furthermore, the instruments were in the same order for all cases.

#### 2.1.4 Phase 3

Convenience sampling was utilized to recruit 347 students from Ferdowsi University of Mashhad from 11 to 26 March 2024. The survey was conducted in an identical manner to the phase 1. Then, 28 cases had unacceptable responses and were omitted (8%). Two univariate and two multivariate outliers were removed, so 315 participants (113 males, 201 females; 1 missing) were retained that aged between 18 and 49 (*M* = 22.50, SD = 3.76; 6 missing). Besides checking internal consistency, four tools were used including the PIMIS, socio-demographic inquiries, and two valid measures to investigate the relationship of neuroticism, openness, and obsessive-compulsive traits with earworm (for construct validity of the PIMIS). Moreover, the questionnaires were applied in the same order for every participant.

### 2.2 Materials

#### 2.2.1 Socio-demographic questions

Demographic questions covered the following variables: education level, mother tongue, English knowledge, economic status, having a job, having religious beliefs, place of residence, only-child status, video gaming background, musicianship, experience of musical training, daily listening to music, reaction to earworms, valence toward stuck songs, earworms language, and recognition of earworms triggers.

#### 2.2.2 Persian involuntary musical imagery scale (PIMIS)

The Involuntary Musical Imagery Scale (IMIS) was developed by Floridou et al. (2015) as a self-report scale encompassing 15 items measuring individual differences in earworm experiences. It was translated and culturally adapted by the authors of the present paper earlier (delineated in phase 0). Persian-IMIS (PIMIS) has four subscales: *Negative Valence*, *Movement, Personal Reflection*, and *Help*. Furthermore, it includes three additional items: one for the frequency, one for the section length, and another for the episode length of earworms. The 15 items are scored on a five-point Likert scale [*1 = Never, 2 = Not very often, 3 = Sometimes, 4 = Most of the time, 5 = Always*]. The frequency question is rated on a six-point Likert scale [*1 = Never, 2 = Once a month, 3 = Once a week, 4 = several times a week, 5 = Several times a day, 6 = Almost continuously*]. The section length and episode length items are scored on a five-point scale separately in terms of duration. The original IMIS had excellent Cronbach’s alpha for the subscales (0.76 to 0.91) and satisfactory test–retest reliability (0.65 to 0.79).

#### 2.2.3 Persian thought control questionnaire (TCQ)

The Thought Control Questionnaire (TCQ) was generated by Wells and Davies (1994) to assess individual differences in the control of unwanted thoughts and validated by Fata et al. (2010) in the non-clinical Persian population. It consists of 29 items scored on a four-point scale (from 1 = almost never to 4 = almost always) that fall into five domains: *Distraction*, *Worry*, *Social control*, *Punishment*, and *Reappraisal*. Items 15, 16, and 17 are scored inversely. The P-TCQ has good Cronbach’s alpha for the subscales (0.64 to 0.74) and the total score (0.91).

#### 2.2.4 Persian depression, anxiety, and stress scale – short form (DASS)

Depression, Anxiety, and Stress Scale (DASS) – short form was built by Lovibond (1995) and adapted for the non-clinical Persian society by Asghari et al. (2008). It has 21 items divided into three factors: *Depression*, *Anxiety*, and *Stress*. Each item is answered on a four-point scale [from 0 = *Never* to 3 = *Almost always*]. The P-DASS showed an excellent Cronbach’s alpha for the subscales (from 0.85 to 0.87). The current study utilized only the two subscales of depression and anxiety.

#### 2.2.5 Persian white bear suppression inventory (WBSI)

The White Bear Suppression Inventory (WBSI; Wegner and Zanakos (1994)) is a 15-item questionnaire that was psychometrically approved for the non-clinical Iranian population (Farrokhi and Mostafapour, 2018) measuring the urge to suppress thoughts. It has one factor, and the items are rated on a five-point scale ranging from 1 (*strongly disagree*) to 5 (*strongly agree*). Higher scores reflect a greater emotional inclination to suppress thoughts. It showed an excellent Cronbach’s alpha (0.87) in Iranian culture.

#### 2.2.6 Persian NEO five-factor inventory (NEO-FFI)

Costa and McCrae (1989) created the NEO Five-Factor Inventory (NEO-FFI). This 60-item instrument quantifies five personality traits (*Neuroticism*, *Openness*, *Agreeableness*, *Conscientiousness*, and *Extraversion*), each of which has 12 items rated on a five-point Likert scale spanning from 0 (*strongly disagree*) to 4 (*strongly agree*). The Persian version was validated in a non-clinical sample of Iranian individuals (Anisi, 2012). Some items have an inverse scoring and demonstrated marginally acceptable to good Cronbach’s alpha (from 0.39 to 0.83). The current study employed only the two subscales of neuroticism and openness.

#### 2.2.7 Persian obsessive-compulsive inventory-revised (OCI-R)

The Obsessive-compulsive Inventory-Revised (OCI-R; Foa et al. (2002)) contains 18 items across six subscales: *Washing*, *Checking*, *Hoarding*, *Ordering*, *Obsessing*, and *Neutralizing*. Each item is evaluated on a five-point scale (from 0 = *Not at all* to 4 = *Extremely*) to measure obsessive and compulsive traits that cause distress. The total score ranges from 0 to 72. The OCI-R was validated in a non-clinical Persian sample (Ghassemzadeh et al., 2011), showing excellent Cronbach’s alpha for subscales (from 0.77 to 0.86) and the overall score (0.85).

### 2.3 Data analysis

Statistical analyses were performed using IBM SPSS 27, LISREL 8.54, and R 4.3.3.

#### 2.3.1 Phase 0

Content validity was assessed using Item-CVI, Item-CVR, and Scale-CVI. Additionally, multi-rater Kappa (Fleiss, 1971) was obtained to show inter-rater reliability.

#### 2.3.2 Phase 1

Exploratory analyses were performed using the following procedures: (1) missing value analysis, (2) normality checking based on skewness and standard error of skewness (*p* < 0.05) along with kurtosis, (3) univariate outlier detection using z-score observations (>3.29, *p* < 0.001) and boxplot, and (4) multivariate outlier detection using MAHALANOBIS distance (*p* < 0.001). For the item analysis, the lower 25% and the upper 25% of the total score were recoded to 1 and 3, respectively. Then, an independent samples *t*-test was used to show the difference between the extreme groups. To check internal consistency, item-total correlations and Cronbach’s alpha (overall and subscales) were obtained. The factorability of PIMIS items was evaluated through an anti-image correlation matrix, Kaiser-Meyer-Olkin measure of sampling adequacy, and Bartlett’s test of sphericity. Factor structure was explored using the Principal Components Analysis (PCA) method along with scree-plots and Varimax rotation. A correlation analysis was conducted between the PIMIS and the Thought Control Questionnaire (TCQ).

#### 2.3.3 Phase 2

Exploratory data analysis was conducted in the same manner as in Phase 1. Multivariate normality was assessed using Mardia’s test with R package (*mvn*) (Korkmaz et al., 2014). The hypothesized model was estimated using the Diagonally Weighted Least Squares method. A correlation analysis was conducted between PIMIS and the two subscales of anxiety and depression from the Depression, Anxiety, and Stress Scale (DASS). Test–retest reliability was obtained using the intraclass correlation coefficient between the responses on PIMIS in phase 2 and the PIMIS at the re-test stage. A correlation analysis was conducted between PIMIS in phase 2 and the White Bear Suppression Inventory (WBSI) at the re-test stage.

#### 2.3.4 Phase 3

Exploratory data analysis was conducted in the same manner as in Phase 1. A correlation analysis was conducted between PIMIS and the Obsessive-compulsive Inventory-Revised (OCI-R) as well as the neuroticism and openness subscales of the NEO Five-Factor Inventory (NEO-FFI).

#### 2.3.5 Further explorations

To delve into individual variations in earworm experiences among the Iranian sample, we combined the data on the PIMIS and demographic questions across the phases 1 to 3. Analyses included tests of Spearman’s correlation as well as two-group comparisons (independent *t*-test; or Mann–Whitney *U* test) and three-group comparisons (ANOVA; or Kruskal Wallis test).

## 3 Results and discussion

### 3.1 Item analysis

In the pilot study, items Q1, Q2, Q5, Q10, Q13, and Q15 were modified due to misleading wording, and item Q17 (episode length) was adapted to Persian syntax to address grammatical ambiguity. There was a weak agreement among experts (*κ* = 0.18, *p* < 0.001), which may be due to the novelty of the explored subject within the area of music psychology. This issue must be taken into consideration in future studies. Moreover, I-CVIs and S-CVI were both excellent (≥0.8 and =0.98, respectively), and I-CVRs were generally acceptable (*≥*0.6) except for two items, Q7 (=0.2) and Q8 (=0.2), which were not practically important due to weak inter-rater reliability. Item analysis revealed a significant difference between extreme groups (*t* = −29.35, df = 159, *p* < 0.001, |*d*| = 4.62). Item-total correlations were generally acceptable except for six items (Q1, Q3, Q5, Q11, Q13, Q15) with *r* < 0.3, which were not problematic due to the low overlap between subscales.

### 3.2 Reliability analysis

On average, Cronbach’s alpha for the overall scale (=0.76) and subscales (*Negative valence* = *0.91*, *Movement* = *0.73*, *Personal reflection* = *0.71*, *Help* = *0.78*) was satisfactory across phases 1 to 3, indicating the PIMIS is internally consistent, consistent with Floridou et al. (2015). Intraclass correlation coefficient for the scale (=0.81) and its subscales (*negative valence* = *0.82*, *movement* = *0.68*, *personal reflection* = *0.74*, and *help* = *0.82*) between the phase 2 and re-test stage suggests that the PIMIS has an excellent test–retest reliability, similar to Floridou et al. (2015).

### 3.3 Factor analysis

**Figure 1:**
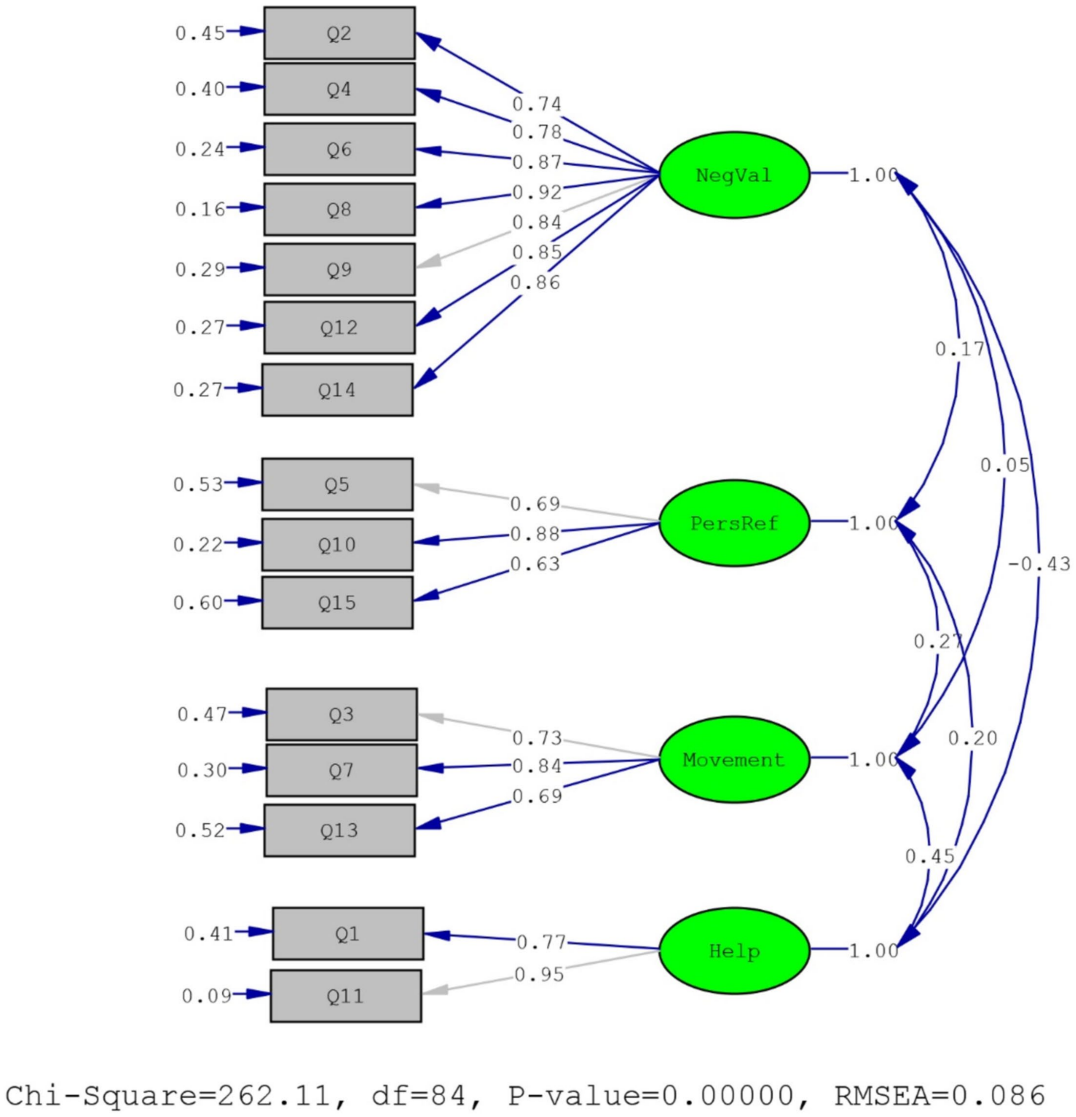
Confirmatory factor analysis of PIMIS using LISREL. NegVal, negative valence; PersRef, personal reflection.

The *KMO* was *0.85*, Bartlett’s test showed a Chi-square of *2106.82* (*df* = 105, *p <* 0.001), and diagonal values of the anti-image correlation matrix (item-KMO) were satisfactory (0.63 to 0.93). The PCA method revealed that communalities were satisfactory (≥0.5), and four factors were extracted with eigenvalues >1, consistent with the scree-plot. Overall, 69.46% of the total variance was explained, and rotated factor loadings were excellent (>0.8), as presented in Table 1. As the multivariate normality was not met (*skewness* = 913.99, *Kurtosis =* 4.26), robust estimation was used (Satorra and Bentler, 2001). Goodness-of-fit indices were excellent ($\chi^{2}$=262.11, *p*-value *= 0.00*, χ2df*=3.12, RMSEA* = 0.08, NFI = 0.96, *CFI* = 0.97, *SRMR* = 0.07, *GFI* = 0.98), $R^{2}$ coefficients for items were generally satisfactory (0.40 to 0.91), and factor loadings were excellent (0.63 to 0.95; as detailed in Figure 1), so the Persian IMIS has a four-factor structure as of Floridou et al. (2015).

**Table 1:** Component matrix after Varimax rotation.

	Factor 1	Factor 2	Factor 3	Factor 4
Q2	0.74			
Q4	0.81			
Q6	0.82			
Q8	0.86			
Q9	0.75			
Q12	0.80			
Q14	0.79			
Q3		0.85		
Q7		0.83		
Q13		0.72		
Q5			0.84	
Q10			0.72	
Q15			0.70	
Q1				0.85
Q11				0.83

### 3.4 Validity analysis

The convergent validity of PIMIS in relation to the Thought Control Questionnaire (TCQ) is summarized in Table 2. It yielded a correlation of the TCQ total score with PIMIS negative valence and personal reflection, consistent with “cognitive attentional syndrome” (CAS), suggesting that concentration on unwanted thoughts (e.g., INMI) would result in more distress. Inversely, the association between TCQ reappraisal and PIMIS help is in line with Wells (2009) notion that how we think about our thoughts does matter. Moreover, the correlation of TCQ punishment with PIMIS negative valence and help (inverse), plus the correlation of TCQ worry with PIMIS negative valence and personal reflection, supports the mediational role of impaired mental control (Wells and Davies, 1994). Additionally, TCQ punishment and worry were associated with PIMIS episode length, suggesting that episode length might be a central element of earworms’ intrusiveness. TCQ social control was found to be related to the PIMIS movement, conforming with the tenet of Tarr et al. (2014) in terms of the social bonding nature of music. Finally, the inverse relationship between TCQ distraction and PIMIS personal reflection advocates the finding suggested by Wegner (1989) that distraction effectively reduces unwanted thoughts under some conditions.

**Table 2:** Correlation analysis of Persian IMIS with TCQ, DASS, WBSI, NEO-FFI, and OCI-R.

	Negative valence	Movement	Personal reflection	Help	Frequency	Section length	Episode length
TCQ							
Distraction	−0.03	−0.05	−0.13^*^	0.005	0.01	0.01	−0.10
Worry	0.14^*^	−0.03	0.24^***^	0.05	0.006	−0.01	0.11^*^
Social control	−0.03	0.12^*^	0.04	−0.01	0.02	−0.02	−0.03
Punishment	0.30^***^	0.16^**^	0.21^***^	−0.12^*^	0.08	−0.03	0.17^**^
Reappraisal	−0.02	0.19^***^	0.01	0.14^*^	0.03	0.08	0.06
Total score	0.12^*^	0.17^**^	0.13^*^	0.02	0.04	0.009	0.08
DASS							
Depression	0.08	−0.01	0.14^*^	−0.02	0.14^*^	0.02	0.07
Anxiety	0.18^**^	0.07	0.26^***^	−0.02	0.008	0.05	0.08
WBSI							
Total score	0.11	0.11	0.09	0.13	0.31^*^	0.21	0.16
NEO-FFI							
Neuroticism	0.18^***^	−0.02	0.36^***^	0.01	0.07	0.004	0.19^***^
Openness	−0.08	0.08	0.13^*^	0.12^*^	0.11	−0.07	0.01
OCI-R							
Washing	0.10	0.16^**^	0.17^**^	0.15^**^	0.11	0.10	0.06
Checking	0.13^*^	0.06	0.22^***^	0.03	0.08	0.10	0.05
Ordering	0.20^***^	0.07	0.26^***^	0.03	0.15^**^	0.09	0.04
Obsessing	0.12^***^	0.03	0.34^***^	0.04	0.11	−0.005	0.19^***^
Hoarding	0.15^**^	0.11^*^	0.29^***^	0.07	0.06	0.07	0.08
Neutralizing	0.12^*^	0.07	0.24^***^	0.06	0.07	0.07	0.08
Total score	0.19^***^	0.11^*^	0.34^***^	0.09	0.13^*^	0.10	0.12^*^

*p < 0.05 (two-tailed); **p < 0.01 (two-tailed); ***p < 0.001 (two-tailed). All correlations are obtained using Spearman’s correlation coefficient.

The concurrent validity of PIMIS in relation to the Depression, Anxiety, and Stress Scale (DASS) is summarized in Table 2. It has shown that depression was correlated with INMI frequency but not negative valence, inconsistent with Ulor et al. (2022) finding on positive association between depressive symptoms and negative valence. Conversely, anxiety was correlated with negative valence toward INMI but not frequency. This double dissociation of results might be interpreted through the correlation of PIMIS personal reflection with depression and anxiety, i.e., depressive individuals may be hypo-sensitive to personalized earworms due to low mood, and anxious individuals could be hyper-sensitive to personalized earworms due to an irritable cognitive state.

The predictive validity of PIMIS in relation to the White Bear Suppression Inventory (WBSI) is summarized in Table 2. A positive correlation between WBSI total score and INMI frequency supports Mullensiefen et al. (2014) notion that trying to suppress thoughts may lead to increased occurrence, evident in the “Ironic Mental Control Theory” (Wegner, 1994).

The construct validity in relation to the NEO Five-Factor Inventory and Obsessive-compulsive Inventory-Revised is summarized in Table 2. Correlation analysis of PIMIS in relation to Big Five personality traits showed that INMI frequency was not associated with neuroticism and openness, in the same line as some previous research (Floridou et al., 2012; Jue et al., 2022) but contrary to others that attained positive correlation of INMI frequency with neuroticism and openness (Williamson and Müllensiefen, 2012; Cotter et al., 2016; Negishi and Sekiguchi, 2020). No relationship of openness was found with PIMIS movement, section length, and episode length, which is inconsistent with Jue et al. (2022) that found positive correlations of openness with PIMIS movement, section length, and episode length. Neuroticism was not only correlated with PIMIS negative valence, consistent with Jue et al. (2022), but also with INMI episode length for the first time among Asian countries. Another novel finding in our data in Asian societies was the association between openness and PIMIS help, that confirms the previous literature (Ullah, 2017; Gutiérrez et al., 2005) that individuals high on openness have a positive attitude toward new [unwanted] experiences including INMI. Correlation analysis of PIMIS in relation to Obsessive-Compulsive (OC) personality traits demonstrated that higher OC traits are correlated with PIMIS negative valence and frequency, which is consistent with Mullensiefen et al. (2014) and Floridou et al. (2015). Furthermore, we found a positive association of higher OC traits with PIMIS episode length, movement, and negative valence, which replicates Jue et al. (2022) findings. OCI obsessing was also correlated with negative valence and episode length, confirming the critical role of episode length in the intrusiveness of earworms.

### 3.5 Multifaceted investigation of earworms

#### 3.5.1 Demographic descriptives

Most people (75.3%) reported that they choose “let it be” in reaction to earworms, which supports previous notion that people around the world prefer to be passive toward earworm experiences (Williamson et al., 2014; Liikkanen and Jakubowski, 2020). Further details about descriptive statistics for other demographic factors are shown in Table 3.

**Table 3:** Demographic Descriptive Statistics (n = 946).

	*N* (%)	Missing *N* (%)
Education level		
Bachelor	689 (72.8%)	2 (0.2%)
Master	172 (18.2%)	
PhD/postdoc	83 (8.8%)	
Mother tongue		
Persian	914 (96.6%)	4 (0.4%)
Non-Persian	28 (3%)	
English knowledge		
Yes	864 (91.3%)	5 (0.5%)
No	77 (8.1%)	
Economic status		
Lower than average	97 (10.3%)	5 (0.5%)
Average	620 (65.5%)	
Higher than average	224 (23.7%)	
Having a job		
Yes	278 (29.4%)	3 (0.3%)
No	665 (70.3%)	
Having religious beliefs		
Yes	703 (74.3%)	8 (0.8%)
No	235 (24.8%)	
Place of residence		
Urban	910 (96.2%)	5 (0.5%)
Rural	31 (3.3%)	
Only-child		
Yes	80 (8.5%)	5 (0.5%)
No	861 (91%)	
Videogaming background		
Gamer	88 (9.3%)	3 (0.3%)
Having experience of videogaming	642 (67.9%)	
No experience of videogaming	213 (22.5%)	
Musicianship		
Yes	115 (12.2%)	4 (0.4%)
No	827 (87.4%)	
Having experience of musical training		
Yes	227 (24%)	6 (0.6%)
No	713 (75.4%)	
Daily listening to music		
Yes	652 (68.9%)	3 (0.3%)
No	291 (30.8%)	
Reaction to earworm experience		
Trying to stop	232 (24.5%)	2 (0.2%)
Let it be	712 (75.3%)	
Valence toward stuck songs		
Liked music	320 (33.8%)	4 (0.4%)
Disliked music	39 (4.1%)	
Both	583 (61.6%)	
Earworms language		
Persian	309 (32.7%)	5 (0.5%)
Non-Persian	34 (3.6%)	
Both	598 (63.2%)	
Recognizing earworms triggers		
Yes	365 (38.6%)	3 (0.3%)
No	578 (61.1%)	

#### 3.5.2 Earworm descriptives

It was found that 25.8% of Iranian university students experience earworms daily, which is lower than in African culture (Pitman et al., 2023) but higher than in the Chinese population (Jue et al., 2022). Likewise, 82.9% reported earworms at least once a week, which is lower than in African (Pitman et al., 2023) and European/American society (Liikkanen, 2012b) but higher than in Chinese people (Jue et al., 2022). These differences might result from musical preferences in various cultures as well as some cultural-religious restrictions that exist in Iranian culture. More detailed descriptions of other aspects of earworms are presented in Table 4.

**Table 4:** Earworm descriptives (*n* = 946).

	*N* (%)	Missing *N* (%)
Frequency		
Never	–	20 (2.1%)
Once a month	142 (15%)	
Once a week	176 (18.6%)	
Several times a week	364 (38.5%)	
Several times a day	165 (17.4%)	
Almost continuously	79 (8.4%)	
Section length		
Less than 5 s	63 (6.7%)	1 (0.1%)
Between 5 and 10 s	321 (33.9%)	
Between 10 and 30 s	273 (28.9%)	
Between 30 s and 1 min	159 (16.8%)	
More than 1 min	129 (13.6%)	
Episode length		
Less than 10 min	418 (44.2%)	0 (0%)
Between 10 min and half an hour	248 (26.2%)	
Between half an hour and 1 h	126 (13.3%)	
Between 1 and 3 h	90 (9.5%)	
More than 3 h	64 (6.8%)	

#### 3.5.3 Inter-correlation of PIMIS items

Data indicates more frequent earworms are correlated with longer sections and episodes along with more helpfulness [but not negative valence], suggesting that INMI is a pleasant phenomenon in our sample, which corroborates the notion that earworm is not considered aversive (Halpern and Bartlett, 2011; Liikkanen, 2012b; Liikkanen and Jakubowski, 2020; Beaman and Williams, 2010; Floridou et al., 2015). Likewise, the negative correlation between PIMIS negative valence and help contradicts Floridou et al. (2015) tenet of the “duality” of earworms’ usefulness-disturbance. Furthermore, only episode length was positively correlated with PIMIS negative valence (among PIMIS additional items), similar to previous evidence (Mullensiefen et al., 2014; Floridou et al., 2015), indicating that episode length might be a principal factor in INMI aversiveness. Detailed correlations are summarized in Table 5.

**Table 5:** Correlation analysis of PIMIS elements.

PIMIS	Frequency	Section length	Episode length	Negative valence	Movement	Personal reflection	Help
Frequency	–	0.22^***^	0.19^***^	−0.04	0.19^***^	0.20^***^	0.21^***^
Section length	–	–	0.24^***^	−0.01	0.07^*^	0.12^***^	0.13^***^
Episode length	–	–	–	0.15^***^	0.08^*^	0.10^**^	0.008
Negative valence	–	–	–	–	−0.007	0.14^***^	−0.38^***^
Movement	–	–	–	–	–	0.20^***^	0.28^***^
Personal reflection	–	–	–	–	–	–	0.15^***^
Help	–	–	–	–	–	–	–

*p < 0.05 (two-tailed); **p < 0.01 (two-tailed); ***p < 0.001 (two-tailed). All correlations are obtained using Spearman’s correlation coefficient.

#### 3.5.4 Individual differences in earworms experiences

To investigate individual variations in relation with demographic variables in Iranian context, demographic-related earworm experiences were probed through group-wise comparisons as follows.

##### 3.5.4.1 Gender

No gender effect was found in INMI frequency (*U* = 79795.5, *p* = 0.16), and earworm section length (U = 84,289, *p* = 0.35), which is consistent with previous research (Beaman and Williams, 2010; Jue et al., 2022; Beaman and Williams, 2013). Nevertheless, females’ INMI episodes were longer than that of males (*U* = 80,237, *p* < 0.05, *r_equivalent_* = 0.06), which is congruent with Pitman et al. (2023). Moreover, females’ negative valence toward earworms was higher than males (*t* = 4.51, *df =* 916, *p* < 0.001, |*d*| = 0.33), which might be explained by their higher susceptibility to affective problems (Young et al., 1990).

##### 3.5.4.2 Age

Age was not associated with the PIMIS negative valence (*p* = 0.49), and earworm episode length (*p* = 0.29), which is inconsistent with Beaman and Williams (2010) that found negative correlation between age and INMI negative valence. However, age was negatively correlated with INMI section length (ρ = −0.08, *p* < 0.01), which is congruent with Pitman et al. (2023). Likewise, Earworms diminish as the age increases (ρ = −0.12, *p <* 0.001), similar to Floridou et al. (2019), highlighting the role of musical engagement as musical involvement decreases at higher ages (Bonneville-Roussy et al., 2013). Consequently, further research on the relationship between musicality and age in Asian countries are recommended.

##### 3.5.4.3 Education level

Earworm frequency was higher in the PhD/postdoc group than in the bachelor’s, and in the bachelor’s group higher than in the master’s group (*H* = 7.90, *df* = 2, *p* < 0.05, η^2^ = 0.006). As the master’s degree is an intensive level in Iran’s educational system, this difference might imply an inverse relationship between cognitive load and INMI frequency, which is concordant with Floridou et al. (2017). However, no significant difference was found between the education levels in earworm section length (*H* = 0.21, df = 2, *p* = 0.81) or episode length (*H* = 1.11, df = 2, *p* = 0.57), which could imply that characteristic earworm experiences proceed through distinct mechanisms, consistent with Floridou et al. (2015).

##### 3.5.4.4 Having a job

Although there was no significant difference between the groups in earworm frequency (*U* = 88805.5, *p* = 0.97) and episode length (*U* = 90,659, *p* = 0.62), participants with a job experienced longer sections of earworms than those without a job.(*U =* 80,161, *p <* 0.001, *r*_equivalent_ = 0.10). Mixed results regarding the relationship of cognitive load with *education level* and *having a job* suggest the multifaceted association between INMI and cognitive load, which would be studied under highly controlled laboratory settings.

##### 3.5.4.5 English knowledge

The English-familiar group had higher earworm frequency (*U* = 26440.5, *p* < 0.05, *r*_equivalent_ = 0.08), and longer earworm sections (*U* = 28,086, *p* < 0.05, *r*_equivalent_ = 0.07) and episodes (*U* = 28,210, *p* < 0.05, *r*_equivalent_ = 0.07) compared to the English-unfamiliar group. These findings are in contrast with that of Pitman et al. (2023) that stated individuals with English home language report shorter sections of earworm. As the English language is not a first language in Iran and Africa, these contradictory results imply that more comprehensive studies are needed to show the effects of bilingualism on earworm experiences.

##### 3.5.4.6 Having religious beliefs

Although no significant difference was explored between the groups in earworm frequency (*U* = 76744.5, *p* = 0.66), section length (*U* = 78017.5, *p* = 0.19), the earworm episodes for the group without religious beliefs were longer than for those with religious beliefs (*U* = 73062.5, *p* < 0.01, *r*_equivalent_ = 0.09), which is inconsistent with Bailes (2015) emphasis on more frequent earworms in people sensitive to inner reflections. This discrepancy suggests that additional studies are required to unravel this topic using standardized religiousness measures.

##### 3.5.4.7 Place of residence

No significant distinction was found between the groups in earworm frequency (*U* = 13,525, *p =* 0.84), section length (*U* = 12945.5, *p* = 0.42), and episode length (*U* = 12910.5, *p* = 0.39). These findings are different from those of Jue et al. (2022) that points out urban-residing people experience more earworms than rural-residing individuals. This inconsistency might result from distinct social factors between the Asian countries might explain.

##### 3.5.4.8 Only-child status

No significant contrast was found between the groups in earworm frequency (*U* = 29,769, *p* = 0.20), section length (*U* = 32,615, *p =* 0.42), and episode length (*U* = 34276.5, *p* = 0.94). These findings are in opposition to those of Jue et al. (2022) that declares the frequency of earworms in the only-child group is more than others with siblings. This contradiction may be due to the different cultural backgrounds of the Asian countries.

##### 3.5.4.9 Videogaming background

No significant difference was found between the groups in earworm section length (H = 3.61, df = 2, *p* = 0.16) and episode length (H = 4.56, df = 2, *p* = 0.10). However, the gamers had a higher earworm frequency than those having experience and those without videogaming experience (*H* = 22.03, *df* = 2, *p* < 0.001, η^2^ = 0.02), which corroborates previous studies (Ortiz De Gortari, 2018; Floridou et al., 2022). Moreover, videogaming status had a significant effect on the helpfulness of earworm experiences (*F* = 7.44, *df* = 2, *p* < 0.001, *η^2^* = 0.02), i.e., the Tucky HSD test revealed no significant difference between the group having experience and the group with no experience (*p* = 0.87), but the gamers reported significantly higher helpfulness of earworms than those having experience and those with no experience (for both, *p* < 0.001). This finding supports evidence from that of Achtman et al. (2008). Overall, it appears the association between videogaming background and aspects of earworms might be the same across different cultures.

##### 3.5.4.10 Musicianship

No difference was found between musicians and non-musicians in earworm frequency (*U* = 41498.5, *p* = 0.12), which is inconsistent with previous literature (Levitin, 2006; Liikkanen, 2012b) that believes musicians report more frequent earworms than non-musician. Likewise, musicianship had no effect on negative valence toward earworms (*t* = −1.08, *df* = 914, *p* = 0.28), which is incompatible with that of Hyman et al. (2013) that states musicians report more negative valence towards earworms than non-musician. However, musicians reported significantly longer earworm sections (U *=* 39,920, *p* < 0.01, *r*_equivalent_ = 0.09) but not episode length (*U* = 42616.5, *p =* 0.056), compared to non-musicians. Generally, various aspects of earworms in musical expertise function distinctly across different cultures, suggesting the special need for the utilization of systematic assessments for musicians.

##### 3.5.4.11 Having experience of musical training

No difference was found between the groups in earworm frequency (U = 70,960, *p* = 0.058), section length (U = 78045.5, p = 0.42). However, the group with musical training had only longer earworm episodes than those without experience (*U* = 73807.5, *p <* 0.05, *r*_equivalent_ = 0.06), which is inconsistent with Jue et al. (2022) that found no such difference.

##### 3.5.4.12 Daily listening to music

No significant distinction was obtained between the groups in section length (*U* = 87381.5, *p* = 0.054). However, the daily listeners reported higher earworm frequency (*U* = 76238.5, *p* < 0.001, *r*_equivalent_ = 0.13) and longer earworm episodes (*U* = 86,634, *p* < 0.05, *r*_equivalent_ = 0.07) than the non-daily listeners. This finding is concordant with Jue et al. (2022), illustrating that daily listeners possess special capabilities to adapt to their everyday earworms.

##### 3.5.4.13 Reaction to earworm experience

The group trying to let it be reported a higher frequency of earworms (*U* = 69,122, *p* < 0.01, r_equivalent_ = 0.09) and found them more helpful (*t* = −11.58, df = 458.97, p < 0.001, |d| = 0.80). Conversely, the individuals trying to stop earworms had longer earworm episodes than those trying to let it be (*U* = 73,027, *p* < 0.01, *r*_equivalent_ = 0.09) and exhibited a higher negative valence toward earworms (*t* = 24.59, *df =* 915, *p* < 0.001, |*d*| = 1.88). These results replicate Wegner (1994) “ironic mental control theory.”

##### 3.5.4.14 Valence toward stuck songs

No significant difference was found between the groups in earworm episode length (*H* = 4.08, df = 2, *p* = 0.13). However, the “both” group had a higher frequency of earworms than the “liked” group, and the “liked” group higher than the “disliked” group (*H* = 11.41, *df* = 2, *p* < 0.01, *η^2^* = 0.01). Nevertheless, the “liked” group had a longer earworm section than the “both” group, and the “both” group longer than the “disliked” (*H* = 11.51, *df* = 2, *p* < 0.01, η^2^ = 0.01). Valence toward stuck songs had a significant effect on negative valence toward earworms (*F* = 33.31, *df* = 2, *p* < 0.001, *η^2^* = 0.06), i.e., the Tucky HSD test demonstrated that the “disliked” group reported higher negative valence than the “both” group (*p* < 0.001), the “both” higher than the “liked” (*p* < 0.001), and the “disliked” higher than the “liked” group (*p* < 0.001). Moreover, Valence toward stuck songs had a significant effect on earworm helpfulness (*F* = 14.02, *df* = 2, *p <* 0.001, η^2^ = 0.02), i.e., the Tucky HSD test revealed the “liked” group found earworms more helpful than the “both” group (*p* < 0.01), the “both” more than the “disliked” (*p* < 0.001), and the “liked” more than the “disliked” (*p* < 0.001). Given a greater frequency, section length, and helpfulness for earworms of liked music than those of disliked music are advocated by previous evidence (Beaman and Williams, 2010; Liikkanen, 2012b; Hyman et al., 2015). This evidence provides confirmatory data on the enjoyable nature of INMI.

##### 3.5.4.15 Earworm language

No significant distinction was found between the groups in earworm episode length (*H* = 5.69, df = 2, *p* = 0.06). However, the “non-Persian” earworms had higher earworm frequency (*H* = 35.27, *df* = 2, *p* < 0.001, *η^2^* = 0.03) and section length (*H* = 8.38, *df* = 2, *p* < 0.05, *η^2^* = 0.006) than the “both,” and the “both” higher than the “Persian,” which is contrary to Liikkanen (2012b) note that language and lyrics does not matter in musical imagery. It is unclear whether this difference is due to different genres or special features of the Persian language, which requires further studies.

##### 3.5.4.16 Recognizing earworm triggers

Most participants (61.1%) reported that their earworm triggers are often unidentifiable, which refutes the previous literature that earworm triggers are often recognizable (Williamson et al., 2012; Williamson et al., 2014). There was no significant difference between the groups in earworm frequency (*U* = 94827.5, *p* = 0.10), section length (*U* = 102345.5, *p* = 0.46), and episode length (*U* = 98946.5, *p* = 0.08). By contrast, the individuals who often do not recognize their earworm triggers tend to find their earworms more intrusive than those who recognize them (*t* = −2.12, *df* = 788.04, *p <* 0.05, |*d*| = 0.14). This finding could be explained by the uncontrollability of thoughts as a result of “ruminative style” (Akerman-Nathan et al., 2024).

##### 3.5.4.17 Mother tongue

No significant difference was obtained between Persians and non-Persians in earworm frequency (*U* = 11504.5, *p* = 0.65), section length (11567.5, *p* = 0.37), and episode length (*U* = 12,751, *p* = 0.97). Compared with the results from association between earworm and *English knowledge* in our paper, it could be stated that although earworms could be closely related to more complex and dynamic aspects of language, the current finding on association between earworm and *Mother tongue* implies that this relationship might not exist for simpler and more constant forms of language.

##### 3.5.4.18 Economic status

No significant disparity was found between different economic conditions in earworm frequency (H = 1.18, df = 2, *p* = 0.55), section length (H = 0.04, df = 2, *p* = 0.98), and episode length (H = 0.02, df = 2, *p* = 0.99). This finding could imply that economic constraint or richness does not affect the availability and/or selection of songs, and therefore the characteristic earworm experiences.

## 4 Conclusion

The objective of this study was to assess the psychometric features of the Persian Involuntary Musical Imagery Scale (PIMIS) alongside multi-dimensional exploration of earworms in a sample of Iranian college students. It was found that PIMIS is a valid and reliable instrument for measuring individual variations of earworms in Iranian context. Exploiting various questionnaires in different validation stages along with coverage of various demographic variables using a large sample has provided a relatively broad outline of Persian-INMI which would be a prominent advantage of the current study. Nevertheless, there are some limitations throughout the study that should be considered fairly. (1) Although using a sample size of almost 300 (Guadagnoli and Velicer, 1988; Comrey, 1988) in different phases provided enough credence to the obtained results, it is noteworthy that most statistical tests were non-parametric, and using a sample of college students might limit the generalization of results to the Iranian population (WEIRD bias). (2) The musical genre of earworms was not investigated. (3) Most demographic variables were measured on a dichotomous scale, which constrains the variation of responses and provides non-systematic evidence. (4) Personality factors were still limited because there were some related factors in the literature, such as schizotypy. It deserves consideration that for the first time this phenomenon was investigated in Iranian context which in turn demonstrated even some contradictory results with other Asian societies. Moreover, INMI is a multifaceted subjective phenomenon, and evidence from large-scale phenomenology and individual differences studies should be exploited as spotlights to discover hidden sides of earworms through meticulous experimentations. Hence, we believe that further cross-cultural validations of IMIS and exploration of the INMI dynamics in other Asian societies would still be valuable. It is obvious that our study was not exhaustive, but it would be a fertile ground for the flourishing of the body of earworm literature in the multicultural and multilingual society of Iran.

## Data Availability

The raw data supporting the conclusions of this article will be made available by the authors, without undue reservation.

## References

[ref1] Achtman R. L. Green C. S. Bavelier D. (2008). Video games as a tool to train visual skills. Restor. Neurol. Neurosci. 26, 435–446. doi: 10.3233/RNN-2008-00424, PMID: 18997318 PMC2884279

[ref2] Akerman-Nathan A. Naftalovich H. Kalanthroff E. (2024). The aversiveness of intrusiveness: evidence from involuntary musical imagery. J. Clin. Psychol. 80, 110–126. doi: 10.1002/jclp.23596, PMID: 37716016

[ref3] Anisi J. (2012). Validity and reliability of NEO five-factor inventory (NEO-FFI) on university students. Int. J. Behav. Sci. 5, 351–355.

[ref4] Asghari A. Saed F. Dibajnia P. (2008). Psychometric properties of the depression anxiety stress Scales-21 (DASS-21) in a non-clinical Iranian sample. Int. J. Psychol. 2, 82–102.

[ref5] Bailes F. (2007). The prevalence and nature of imagined music in the everyday lives of music students. Psychol. Music 35, 555–570. doi: 10.1177/0305735607077834

[ref6] Bailes F. (2015). Music in mind? An experience sampling study of what and when, towards an understanding of why. Psychomusicology 25, 58–68. doi: 10.1037/pmu0000078

[ref7] Baruss I. Wammes M. (2009). Characteristics of spontaneous musical imagery. J. Conscious. Stud. 16, 37–61.

[ref8] Beaman C. P. Williams T. I. (2010). Earworms (stuck song syndrome): towards a natural history of intrusive thoughts. Br. J. Psychol. 101, 637–653. doi: 10.1348/000712609X479636, PMID: 19948084

[ref9] Beaman C. P. Williams T. I. (2013). Individual differences in mental control predict involuntary musical imagery. Music. Sci. 17, 398–409. doi: 10.1177/1029864913492530

[ref10] Beaty R. E. Burgin C. J. Nusbaum E. C. Kwapil T. R. Hodges D. A. Silvia P. J. (2013). Music to the inner ears: exploring individual differences in musical imagery. Conscious. Cogn. 22, 1163–1173. doi: 10.1016/j.concog.2013.07.006, PMID: 24021845

[ref11] Bennett S. (2003). Song stuck in your thoughts? Profiling musical imagery repetition (MIR). Society for Music Perception and Cognition Conference

[ref12] Boateng G. O. Neilands T. B. Frongillo E. A. Melgar-Quiñonez H. R. Young S. L. (2018). Best practices for developing and validating scales for health, social, and behavioral research: a primer. Front. Public Health 6:149. doi: 10.3389/fpubh.2018.00149, PMID: 29942800 PMC6004510

[ref13] Bonneville-Roussy A. Rentfrow P. J. Xu M. K. Potter J. (2013). Music through the ages: trends in musical engagement and preferences from adolescence through middle adulthood. J. Pers. Soc. Psychol. 105, 703–717. doi: 10.1037/a0033770, PMID: 23895269

[ref14] Campbell S. M. Margulis E. H. (2015). Catching an earworm through movement. J New Music Res. 44, 347–358. doi: 10.1080/09298215.2015.1084331

[ref15] Comrey A. L. (1988). Factor-analytic methods of scale development in personality and clinical psychology. J. Consult. Clin. Psychol. 56, 754–761. doi: 10.1037/0022-006X.56.5.754, PMID: 3057010

[ref16] Costa P. Mccrae R. (1989). NEO five-factor inventory (NEO-FFI). Odessa, FL: Psychological Assessment Resources, 3.

[ref17] Cotter K. N. Christensen A. P. Silvia P. J. (2016). Musical minds: personality, schizotypy, and involuntary musical imagery. Psychomusicology 26, 220–225. doi: 10.1037/pmu0000158

[ref18] Cotter K. N. Silvia P. J. (2017). Measuring mental music: comparing retrospective and experience sampling methods for assessing musical imagery. Psychol. Aesthet. Creat. Arts 11, 335–343. doi: 10.1037/aca0000124

[ref19] Farrokhi H. Mostafapour V. (2018). Investigating factor structure, validity and reliability of the Persian form of AnxiousThoughts inventory (AnTI), thought control questionnaire (TCQ) and white bear suppression inventory (WBSI) in the clinical population. J. Anal. Cognit. Psychol. 9, 19–31.

[ref20] Farrugia N. Jakubowski K. Cusack R. Stewart L. (2015). Tunes stuck in your brain: the frequency and affective evaluation of involuntary musical imagery correlate with cortical structure. Conscious. Cogn. 35, 66–77. doi: 10.1016/j.concog.2015.04.020, PMID: 25978461

[ref21] Fata L. Moutabi F. Moloudi R. Ziayee K. (2010). Psychometric properties of Persian version of thought control questionnaire and anxious thought inventory in Iranian students. Journal of Psychological models and methods.

[ref22] Fleiss J. L. (1971). Measuring nominal scale agreement among many raters. Psychol. Bull. 76, 378–382. doi: 10.1037/h0031619

[ref23] Floridou G. A. Halpern A. R. Williamson V. J. (2019). Age-related changes in everyday forms of involuntary and voluntary cognition. PsyArXiv.

[ref24] Floridou G. A. Mullensiefen D. (2015). Environmental and mental conditions predicting the experience of involuntary musical imagery: an experience sampling method study. Conscious. Cogn. 33, 472–486. doi: 10.1016/j.concog.2015.02.012, PMID: 25800098

[ref25] Floridou G. A. Peerdeman K. J. Schaefer R. S. (2022). Individual differences in mental imagery in different modalities and levels of intentionality. Mem. Cogn. 50, 29–44. doi: 10.3758/s13421-021-01209-7, PMID: 34462893 PMC8763825

[ref26] Floridou G. A. Williamson V. J. Emerson L. M. (2018). Towards a new methodological approach: a novel paradigm for covertly inducing and sampling different forms of spontaneous cognition. Conscious. Cogn. 65, 126–140. doi: 10.1016/j.concog.2018.07.014, PMID: 30144685

[ref27] Floridou G. A. Williamson V. J. Müllensiefen D. (2012). Contracting earworms: the roles of personality and musicality. Proceed. ICMPC-ESCOM 12, 302–310.

[ref28] Floridou G. A. Williamson V. J. Stewart L. (2017). A novel indirect method for capturing involuntary musical imagery under varying cognitive load. Q. J. Exp. Psychol. 70, 2189–2199. doi: 10.1080/17470218.2016.1227860, PMID: 27557154

[ref29] Floridou G. A. Williamson V. J. Stewart L. Müllensiefen D. (2015). The involuntary musical imagery scale (IMIS). Psychomusicology 25, 28–36. doi: 10.1037/pmu0000067

[ref30] Foa E. B. Huppert J. D. Leiberg S. Langner R. Kichic R. Hajcak G.. (2002). The obsessive-compulsive inventory: development and validation of a short version. Psychol. Assess. 14, 485–496. doi: 10.1037/1040-3590.14.4.485 12501574

[ref31] Ghassemzadeh H. Shams G. Abedi J. Karamghadiri N. Ebrahimkhani N. Rajabloo M. (2011). Psychometric properties of a Persian-language version of the obsessive-compulsive inventory-revised: OCI-R-Persian. Psychology 2, 210–215. doi: 10.4236/psych.2011.23032

[ref32] Guadagnoli E. Velicer W. F. (1988). Relation of sample size to the stability of component patterns. Psychol. Bull. 103, 265–275. doi: 10.1037/0033-2909.103.2.265, PMID: 3363047

[ref33] Gutiérrez J. L. G. Jiménez B. M. Hernández E. G. Pcn C. (2005). Personality and subjective well-being: big five correlates and demographic variables. Personal. Individ. Differ. 38, 1561–1569. doi: 10.1016/j.paid.2004.09.015

[ref34] Halpern A. R. Bartlett J. C. (2011). The persistence of musical memories: a descriptive study of earworms. Music. Percept. 28, 425–431.

[ref35] Hyman J. R. I. E. Burland N. K. Duskin H. M. Cook M. C. ROY C. M. Mcgrath J. C.. (2013). Going Gaga: investigating, creating, and manipulating the song stuck in my head. Appl. Cogn. Psychol. 27, 204–215. doi: 10.1002/acp.2897

[ref36] Hyman J. R. I. E. Cutshaw K. I. Hall C. M. Snyders M. E. Masters S. A. Au V. S.. (2015). Involuntary to intrusive: using involuntary musical imagery to explore individual differences and the nature of intrusive thoughts. Psychomusicology 25, 14–27. doi: 10.1037/pmu0000075

[ref37] Izadifar M. Formuli A. Isham E. A. Paolini M. (2023). Subjective time perception in musical imagery: an fMRI study on musicians. PsyCh J. 12, 763–773. doi: 10.1002/pchj.677, PMID: 37586874

[ref38] Jue D. Jianping M. Yiduo Y. (2022). Validation of the Chinese involuntary musical imagery scale and its application in mainland China. Music. Sci. 26, 326–338. doi: 10.1177/1029864920948572

[ref39] Korkmaz S. Göksülük D. Zararsiz G. (2014). MVN: an R package for assessing multivariate normality. R J. 6. 151–162.

[ref40] Küssner M. B. Taruffi L. Floridou G. A. Welch G. Cross I. Ockelford A. (2022). Music and mental imagery. London. Routledge (Taylor and Francis Group).

[ref41] Levitin D. J. (2006). This is your brain on music: The science of a human obsession. New York: Penguin.

[ref42] Liikkanen L. A. (2008). “Music in everymind: commonality of nvoluntary musical imagery” in 10th international conference of music perception and cognition (Sapporo, Japan), 1–5.

[ref43] Liikkanen L. A. (2012a). Inducing involuntary musical imagery: an experimental study. Music. Sci. 16, 217–234. doi: 10.1177/1029864912440770

[ref44] Liikkanen L. A. (2012b). Musical activities predispose to involuntary musical imagery. Psychol. Music 40, 236–256. doi: 10.1177/0305735611406578

[ref45] Liikkanen L. A. (2018). Involuntary musical imagery: Everyday but ephemeral. Helsingin yliopisto.

[ref46] Liikkanen L. A. Jakubowski K. (2020). Involuntary musical imagery as a component of ordinary music cognition: a review of empirical evidence. Psychon. Bull. Rev. 27, 1195–1217. doi: 10.3758/s13423-020-01750-7, PMID: 32583211 PMC7704448

[ref47] Liikkanen L. A. Jakubowski K. Toivanen J. M. (2015). Catching earworms on TWITTER: using big data to study involuntary musical imagery. Music. Percept. 33, 199–216. doi: 10.1525/mp.2015.33.2.199

[ref48] Lovibond S. H. (1995). Manual for the depression anxiety stress scales. Sydney Psychol. Found.

[ref49] Moeck E. K. Hyman J. R. Takarangi M. K. (2018). Understanding the overlap between positive and negative involuntary cognitions using instrumental earworms. Psychomusicology 28, 164–177. doi: 10.1037/pmu0000217

[ref50] Mullensiefen D. Fry J. Jones R. Jilka S. Stewart L. Williamson V. J. (2014). Individual differences predict patterns in spontaneous involuntary musical imagery. Music. Percept. 31, 323–338. doi: 10.1525/mp.2014.31.4.323

[ref51] Negishi K. Sekiguchi T. (2020). Individual traits that influence the frequency and emotional characteristics of involuntary musical imagery: an experience sampling study. PLoS One 15:e0234111. doi: 10.1371/journal.pone.0234111, PMID: 32497111 PMC7272041

[ref52] Ortiz De Gortari A. B. (2018). Game transfer phenomena: Origin, development, and contributions to the video game research field. Oxford university press.

[ref53] Pitman M. M. Geffen T. Nettleton P. (2023). Psychometric evaluation of the involuntary musical imagery scale (IMIS) in a south African sample. Music. Sci. 27, 495–509. doi: 10.1177/10298649211028643

[ref54] Satorra A. Bentler P. M. (2001). A scaled difference chi-square test statistic for moment structure analysis. Psychometrika 66, 507–514. doi: 10.1007/BF02296192 PMC2905175 20640194

[ref55] Tarr B. Launay J. Dunbar R. I. (2014). Music and social bonding:“self-other” merging and neurohormonal mechanisms. Front. Psychol. 5:1096. doi: 10.3389/fpsyg.2014.01096 25324805 PMC4179700

[ref56] Ullah F. (2017). Personality factors as determinants of psychological well-being among university students. Int. J. Indian Psychol. 4, 5–16.

[ref57] Ulor M. Bailes F. O’connor D. B. (2022). An investigation into the relationship between musical imagery and anxiety. Imagin. Cogn. Pers. 42, 5–23. doi: 10.1177/02762366221083234

[ref58] Wegner D. M. (1989). White bears and other unwanted thoughts: Suppression, obsession, and the psychology of mental control. New York: Penguin Press.

[ref59] Wegner D. M. Zanakos S. (1994). Chronic thought suppression. J. Pers. 62, 615–640. doi: 10.1111/j.1467-6494.1994.tb00311.x, PMID: 7861307

[ref60] Wegner D. M. (1994). Ironic processes of mental control. Psychol. Rev. 101, 34–52. doi: 10.1037/0033-295X.101.1.34, PMID: 8121959

[ref61] Wells A. (2009). Metacognitive therapy for anxiety and depression. New York: Guilford press.

[ref62] Wells A. Davies M. I. (1994). The thought control questionnaire: a measure of individual differences in the control of unwanted thoughts. Behav. Res. Ther. 32, 871–878. doi: 10.1016/0005-7967(94)90168-6, PMID: 7993332

[ref63] Williamson V. J. Jilka S. R. Fry J. Finkel S. Müllensiefen D. Stewart L. (2012). How do “earworms” start? Classifying the everyday circumstances of involuntary musical imagery. Psychol. Music 40, 259–284. doi: 10.1177/0305735611418553

[ref64] Williamson V. J. Liikkanen L. A. Jakubowski K. Stewart L. (2014). Sticky Tunes: how do people react to involuntary musical imagery? PLoS One 9:e86170. doi: 10.1371/journal.pone.0086170, PMID: 24497938 PMC3908735

[ref65] Williamson V. Müllensiefen D. (2012). Earworms from three angles: Situational antecedents, personality predisposition and the quest for a musical formula. Proceedings of the 12th International Conference on Music Perception and Cognition and the 8th Triennial Conference of the European Society for the Cognitive Sciences of Music, Thessaloniki.

[ref66] Young M. A. Fogg L. F. Scheftner W. A. Keller M. B. Fawcett J. A. (1990). Sex differences in the lifetime prevalence of depression: does varying the diagnostic criteria reduce the female/male ratio? J. Affect. Disord. 18, 187–192. doi: 10.1016/0165-0327(90)90035-7, PMID: 2139063

[ref67] Zeigarnik B. (1927). Über das Behalten von erledigten und unerledigten Handlungen. Psychol. Forsch. 9:1.

