# Supplementary material for: Validation of the Persian Involuntary Musical Imagery Scale alongside multifaceted investigation of earworms among Iranian college students
Source: Front Psychol. 2025 Jan 31;16:1480633. doi: 10.3389/fpsyg.2025.1480633 (PMC11827679; doi:10.3389/fpsyg.2025.1480633)
Supplement: Supplementary file 1 [file Table_1.DOCX]

**Table 1.** Content validity results after expert panel review

| Items | I-CVI | I-CVR |
| --- | --- | --- |
| qA (frequency) | 1.00 | 1.00 |
| q1 | 1.00 | 1.00 |
| q2 | 1.00 | 1.00 |
| q3 | 1.00 | 1.00 |
| q4 | 1.00 | 0.60 |
| q5 | 1.00 | 1.00 |
| q6 | 1.00 | 0.60 |
| q7 | 1.00 | 0.20 |
| q8 | 1.00 | 0.20 |
| q9 | 1.00 | 0.60 |
| q10 | 0.80 | 0.60 |
| q11 | 1.00 | 1.00 |
| q12 | 1.00 | 0.60 |
| q13 | 1.00 | 0.60 |
| q14 | 1.00 | 0.60 |
| q15 | 1.00 | 1.00 |
| q16 (Section Length) | 1.00 | 1.00 |
| q17 (Episode Length) | 1.00 | 1.00 |

I-CVI = item content validity index; I-CVR = item content validity ratio

**Table 2.** Item-total statistics

| Item | Corrected Item-Total Correlation |
| --- | --- |
| Q1 | -0.11 |
| Q2 | 0.49 |
| Q3 | 0.20 |
| Q4 | 0.56 |
| Q5 | 0.27 |
| Q6 | 0.56 |
| Q7 | 0.32 |
| Q8 | 0.57 |
| Q9 | 0.43 |
| Q10 | 0.47 |
| Q11 | -0.11 |
| Q12 | 0.53 |
| Q13 | 0.22 |
| Q14 | 0.53 |
| Q15 | 0.24 |

**Table 3.** Anti-image correlation matrix

|  | q1 | q2 | q3 | q4 | q5 | q6 | q7 | q8 | q9 | q10 | q11 | q12 | q13 | q14 | q15 |
| --- | --- | --- | --- | --- | --- | --- | --- | --- | --- | --- | --- | --- | --- | --- | --- |
| q1 | 0.786 |  |  |  |  |  |  |  |  |  |  |  |  |  |  |
| q2 |  | 0.869 |  |  |  |  |  |  |  |  |  |  |  |  |  |
| q3 |  |  | 0.639 |  |  |  |  |  |  |  |  |  |  |  |  |
| q4 |  |  |  | 0.893 |  |  |  |  |  |  |  |  |  |  |  |
| q5 |  |  |  |  | 0.674 |  |  |  |  |  |  |  |  |  |  |
| q6 |  |  |  |  |  | 0.920 |  |  |  |  |  |  |  |  |  |
| q7 |  |  |  |  |  |  | 0.685 |  |  |  |  |  |  |  |  |
| q8 |  |  |  |  |  |  |  | 0.922 |  |  |  |  |  |  |  |
| q9 |  |  |  |  |  |  |  |  | 0.908 |  |  |  |  |  |  |
| q10 |  |  |  |  |  |  |  |  |  | 0.773 |  |  |  |  |  |
| q11 |  |  |  |  |  |  |  |  |  |  | 0.782 |  |  |  |  |
| q12 |  |  |  |  |  |  |  |  |  |  |  | 0.931 |  |  |  |
| q13 |  |  |  |  |  |  |  |  |  |  |  |  | 0.763 |  |  |
| q14 |  |  |  |  |  |  |  |  |  |  |  |  |  | 0.900 |  |
| q15 |  |  |  |  |  |  |  |  |  |  |  |  |  |  | 0.750 |

Diagonal values show measure of sampling adequacy for each item (I-KMO)

**Figure 1.** Scree Plot
